# Pazopanib in advanced desmoplastic small round cell tumours: a multi-institutional experience

**DOI:** 10.1186/2045-3329-4-7

**Published:** 2014-07-29

**Authors:** Anna Maria Frezza, Charlotte Benson, Ian R Judson, Saskia Litiere, Sandrine Marreaud, Stefan Sleijfer, Jean-Yves Blay, Raz Dewji, Cyril Fisher, Winette van der Graaf, Larry Hayward

**Affiliations:** 1 Medical Oncology, University Campus Bio-Medico, Via Alvaro del Portillo 200, Rome 00128, Italy; 2 Sarcoma Unit, Royal Marsden Hospital, London, UK; 3 EORTC HQ, Brussels, Belgium; 4 Medical Oncology, Erasmus MC Cancer Institute, Rotterdam, The Netherlands; 5 Medical Oncology, Centre Leon Berard, Lyon, France; 6 GlaxoSmithKline, Oncology, Uxbridge, UK; 7 Department of Medical Oncology, Radboud University Medical Center, Nijmegen, The Netherlands; 8 Medical Oncology, NHS Lothians, Edinburgh, UK

**Keywords:** Pazopanib, Tyrosine kinase inhibitor, Desmoplastic small round cell tumour

## Abstract

**Background:**

We retrospectively reviewed data from nine pre-treated metastatic desmoplastic small round cell tumour (DSRCT) patients who received pazopanib.

**Patients and methods:**

Three patients received pazopanib within the EORTC phase II 62043, three in the EORTC phase III 62072, and three in the context of UK named patient program.

**Results:**

Nine patients were retrieved from the databases, the median age was 30 years (range: 21–47), they were all males. All had received prior chemotherapy. At the time of treatment start, 4 patients (44%) had ECOG PS 0, 4 (44%) PS 1, 1 (11%) PS 2. Best response was partial response (PR) in 2/9 (22%) patients, stable disease (SD) in 5/9 (56%) and progressive disease (PD) in 2/9 (22%) with a clinical benefit rate (PR + SD > 12 weeks) of 78%. Median PFS and OS were 9.2 (95%CI: 0–23.2) and 15.4 (95%CI: 1.5-29.3) months respectively. With a median follow-up of 20 months, 2/9 (22%) patients are still alive, all progressed. The most common toxicities included neutropenia (G1-2 45%; G3-4 11%), anaemia (G1-2 45%), fatigue (G1-2 67%), diarrhoea (G1-2 45%; G3-4 11%), nausea (G1-2 45%), hypertension (G1-2 45%) and increase in liver enzymes (G1-2 34%; G3-4 11%). Three patients (34%) required a dose reduction. One of the patients discontinued treatment because of persistent increase in total bilirubin level, one due to patient’s choice.

**Conclusion:**

In this series, pazopanib showed interesting activity in DSRCT patients who progressed after prior chemotherapy without major toxicity.

## Background

Pazopanib (GW786034) is an orally available inhibitor of the tyrosine kinases of several factors including the vascular endothelial growth factor receptors (VEGFR) 1–3, c-KIT, and the platelet-derived growth factor receptors (PDGFR) alpha and beta [1]. In addition to advanced clear cell renal cell carcinoma, for which pazopanib received EMA and FDA approval in 2009 [2], pazopanib has been assessed in various other tumor types, including soft tissue sarcoma (STS). Preclinical studies showed that VEGF is over-expressed and that circulating angiogenic factor levels correlate with extent of disease and risk of recurrence in patients with STS [3,4]. Pazopanib activity in STS was initially explored in an EORTC single arm phase II study in patients who failed doxorubicin- and/or ifosfamide-based chemotherapy, stratified by histology (adipocytic STS versus leiomyosarcoma versus synovial sarcoma versus other eligible STS subtypes). In general pazopanib was well tolerated, with hypertension, fatigue, hypopigmentation, and nausea being the most common drug related toxicities, mostly grades (G) 1 to 2. The progression-free survival rate (PFR) at 12 weeks, was 44% for leiomyosarcoma, 49% for synovial sarcoma and 39% for other types of sarcoma. The low PFR for the adipocytic sarcoma stratum (26%) led to the exclusion of this subtype in the subsequent randomised phase III study (PALETTE) [5]. In the PALETTE study 369 patients were randomized to receive pazopanib 800 mg/day versus placebo. Median PFS was 4.6 months (95%CI 3.7–4.8) for pazopanib compared with 1.6 months (95%CI 0.9–1.8) for placebo (P < 0.0001). A positive trend favouring pazopanib was also recorded in overall survival (12.5 months versus 10.7 months), without reaching a statistical significance (P = 0.25) [6]. Patients who had the best chance to benefit with a long survival were those with a good performance status, low or intermediate tumor grade and a normal haemoglobin level at baseline [7]. No differences were recorded according to histology (P = 0.61). Consistently with the data from the phase II study, the most common adverse events were fatigue, diarrhoea, nausea, weight loss and hypertension. Overall, the results of the PALETTE study showed both good tolerability and activity of pazopanib, supporting its value as a new treatment option in advanced non-adipocytic STS patients.

Desmoplastic small round cell tumour (DSRCT) is an unusual subtype of STS, with annual incidence rate of 0.1 case per 1.000.000 [8]. It has distinctive histologic, genetic and clinical features. DSRCT is considered to be a member of the family of small blue round cell tumors and is characterised by a prominent desmoplastic stroma composed of fibroblasts or myofibroblasts embedded in a loose extracellular material; stromal vascularity is also well represented [9]. In 96-97% of all cases, DSRCT harbour a t(11;22) translocation, which involves a fusion of the EWSR1 gene, on chromosome 22, with the WT1 gene, on chromosome 11 [10]. Usually, DSRCT affects young Caucasian males at adolescent and young adult age and typically presents with a widespread involvement of the peritoneal cavity. Crampy abdominal pain and associated palpable abdominal masses are the most common signs at presentation, but abdominal fullness, nausea, constipation and ascites can also be present [11].

Complete surgical resection is the only curative modality, but usually the disease presents late at a stage where complete resection is impossible. Alkylator-based regimens have been proven to be effective in advanced DSRCT, but durable responses are rare [12]. Other novel approaches, such as complete cytoreduction and HIPEC (hyperthermic intraperitoneal chemotherapy) or whole abdominopelvic intensity-modulated radiation therapy (WAP-IMRT) combined with chemotherapy, have also been tested with some promising results [13,14]. However, the value of these observations is limited due to the small sample size and the retrospective nature of the studies. Despite a multimodal approach, the expected 5 years overall survival for DSRCT is only 15% [15].

Preclinical studies have shown that VEGFR-2 and VEGFA are over-expressed in DSRCT and that DSRCT xenografts can be highly responsive to anti-VEGF agents such as bevacizumab [16]. One ongoing pivotal trial is currently assessing the impact of the addition of bevacizumab to a first-line dose dense chemotherapeutic regimen [17]. Moreover, EWSR1/WT1 has been proven to induce the upregulation of PDGF ligand and receptor, which might be responsible for the prominent tumor-associated desmoplasia detected in this disease [18-20]. On this bases, imatinib activity was tested in two phase II studies, showing unfortunately no efficacy in advanced DSRCT (no response reported) [21,22]. Italiano et al. recently reported on their experience in advanced DSRCT patients treated with sunitinib, a multi-kinase inhibitor that blocks several tyrosine kinase receptors such as VEGF receptors, PDGF receptors, KIT, FLT3, and CSF-1. Among eight patients treated, two patients achieved a partial response (25%), three (37.5%) had stable disease, and three (37.5%) had progressive disease. Median progression free survival (PFS) was 2.6 months (95%CI 0–9). Interestingly, one patient was still on treatment 10 months after its initiation [23]. These results suggest that both VEGFR and PDGFR can represent promising targets and that small molecule tyrosine kinase inhibitors (TKIs) blocking these receptors and their downstream pathways might represent a possible treatment option for advanced DSRCT patients. Of note, a recent phase I pharmacokinetic and pharmacodynamic study of pazopanib in children affected by advanced STS or other refractory solid tumors reported a sustained partial response (PR) in a DSRCT patient [24].

Here we describe, for the first time, the activity of pazopanib in nine pre-treated patients with metastatic desmoplastic DSRCT, with the aim of reporting on efficacy and tolerability.

## Methods

### Patients population

We retrospectively reviewed data from nine patients affected by advanced DSRCT progressing on or after prior chemotherapy, comprising three DSRCT patients treated within the EORTC phase II study 62043, three in the EORTC phase III study 62072 (PALETTE), along with three patients treated in the UK on the subsequent pazopanib named patient program (at Royal Marsden Hospital, London, and NHS Lothians, Edinburgh). All cases were reviewed by sarcoma expert pathologists, familiar with the diagnosis of DSRCT.

### Data collection

Details on patient (age at diagnosis, gender, baseline ECOG performance status), disease (primary origin, metastatic sites), treatment (starting dose, dose reduction, toxicities) and outcome (best response according to RECIST 1.0 criteria, progression-free survival and overall survival) were recorded [25]. In the UK data were extracted and analysed from individual patient files, the data from patients in the phase 2 and 3 study were retrieved from the EORTC database in Brussels.

### Statistical analysis

Descriptive analysis was made using median values and range. Progression-free survival (PFS) and overall survival (OS) were estimated with Kaplan-Meier method [26]. PFS was defined as the moment from start of pazopanib until progressive disease according to RECIST, or death. OS was defined as the time from start of pazopanib to death due to any cause. Alive patients were censored at the time of the last contact. SPSS software (version 17.00, SPSS, Chicago, ILQ5) was used for statistical analysis. A P value of less than 0.05 was considered to indicate statistical significance.

## Results

Data from nine patients included in this analysis revealed a median age of 30 years (range: 21–47). All patients were males and were affected by widespread metastatic DSRCT. Four patients had one previous chemotherapy line (44%), four had 2 previous chemotherapy lines (44%) and one patient 3 (12%). At the time of treatment start, four patients (44%) had ECOG PS 0, four (44%) PS 1 and one (11%) PS 2. Pazopanib was started at 800 mg/day in 8/9 patients (89%) while one patient received 600 mg/day due to impaired PS. All patients were evaluable for response. Using RECIST 1.0 criteria, best response was partial response (PR) in 2/9 (22%) patients, stable disease (SD) in 5/9 patients (56%) and progressive disease (PD) in 2/9 (22%) with a clinical benefit rate (PR + SD > 12 weeks) of 78%. These data are summarised in Table 1. Median PFS and OS were 9.2 (95%CI: 0–23.2) and 15.4 (95%CI: 1.5-29.3) months respectively. With a median follow-up of 20 months, 2/9 (22%) patients are still alive, all progressed. The most common haematological toxicities were neutropenia (G1-2 45%; G3-4 11%) and anaemia (G1-2 45%), while non-haematological toxicities included fatigue (G1-2 67%), diarrhoea (G1-2 45%; G3-4 11%), nausea (G1-2 45%), hypertension (G1-2 45%) and increase in liver enzymes (G1-2 34%; G3-4 11%). A dose reduction was required in 3/9 (34%) patients. One patient discontinued treatment because of persistent G3 increase in total bilirubin level, one due to patient’s choice, seven because of progressive disease.

**Table 1:** Pazopanib in advanced desmoplastic small round cell tumour

**Case**	**Age at onset of pazopanib**	**Sex**	**PS**	**Site of metastases**	**Starting dose (mg)**	**Dose reduction**	**Best response**	**PFS (months)**	**OS (months)**
1	21	M	2	Peritoneum, liver, kidney	600	N	PD	0.5	2.1
2	22	M	1	Peritoneum	800	Y	SD	9.2	9.7
3	47	M	1	Peritoneum, liver	800	N	PR	16.3	25.8
4	21	M	1	Peritoneum, liver	800	N	SD	3.9	9.2
5	30	M	0	Peritoneum, lung, lymph nodes, liver	800	Y	PD	2.5	7.4
6	33	M	0	Peritoneum, liver	800	Y	SD	9.4	15.4
7	20	M	1	Peritoneum, lung, bone	800	N	SD	4.4	26
8	30	M	0	Peritoneum, liver, lymphnodes	800	N	SD	9.9	26.7*
9	30	M	0	Peritoneum, liver	800	N	PR	13.1	13.8*

*Still alive.

## Discussion

Here, for the first time, we reported prolonged PFS and OS in metastatic DCRST patients treated with pazopanib and progressing on previous chemotherapy. The observed PFS 9.2 (95%CI: 0–23.2) is remarkable, especially if compared with the survival recently reported on sunitinib (2.6 months) or temsirolimus (9 months, one patient only) and with the one in the PALETTE study, which was 4.6 months only [6,23,27]. The OS in the present series (15.4 months; 95%CI: 1.5-29.3) appears also encouraging as compared to the OS in the PALETTE study (12.5 months) [6].

Previous series showed a survival improvement in DSRCT patients treated upfront with aggressive multimodal approach including the use of a multi-agents alkylator-based first-line regimen (P-6 regimen), surgical debulking, and radiotherapy (3-years survival: 55% vs 27% when all three modalities were not used; P < 0.02) [12,15,28]. However, the use of the P-6 regime is limited by substantial toxicity. More recently, encouraging results have been also reported with the use of standard Ewing sarcoma regimens in the first line (VIDE and VDC/IE), with a median TTP of approximately 15 months and a favourable tolerability profile [29,30]. Two cases in literature suggested the efficacy of trabectedin in DSRCT, which is considered an option in patients with recurrent disease [31,32]. Despite the efforts done, the outcome of DSRCT patients is still miserable: even with a high initial response rate to alkylator-based regimens (90-95%), the median time to progression (TTP) on/after the first-line chemotherapy has been recently reported to be approximately 4 months, the efficacy and regimen of chemotherapy at the time of progression is unproven and the overall median survival is about 16 to 23 months [11,30]. Therefore, the identification of different pathways potentially relevant in the pathogenesis and the development of novel compounds potentially active in the treatment of this aggressive disease is crucial. The oncogenic fusion product EWSR1/WT1 in DSRCT was reported to activate the IGF-1R gene promoter, providing the basis to test the activity of anti-IGF-1R antibodies in the metastatic setting. In a recent phase II study, ganitumab administered in 16 metastatic DSRCT patients determined one PR (6%) and 10 (63%) SD, with a median PFS of 15 months [33]. A different anti-IGF-1R antibody, cixutumumab, has been recently tested in a phase I study in combination with temsirolimus, an m-TOR inhibitor which efficacy in DSRCT has been previously described in a case report by Thijs A. et al. [27,34].

**Figure 1:**
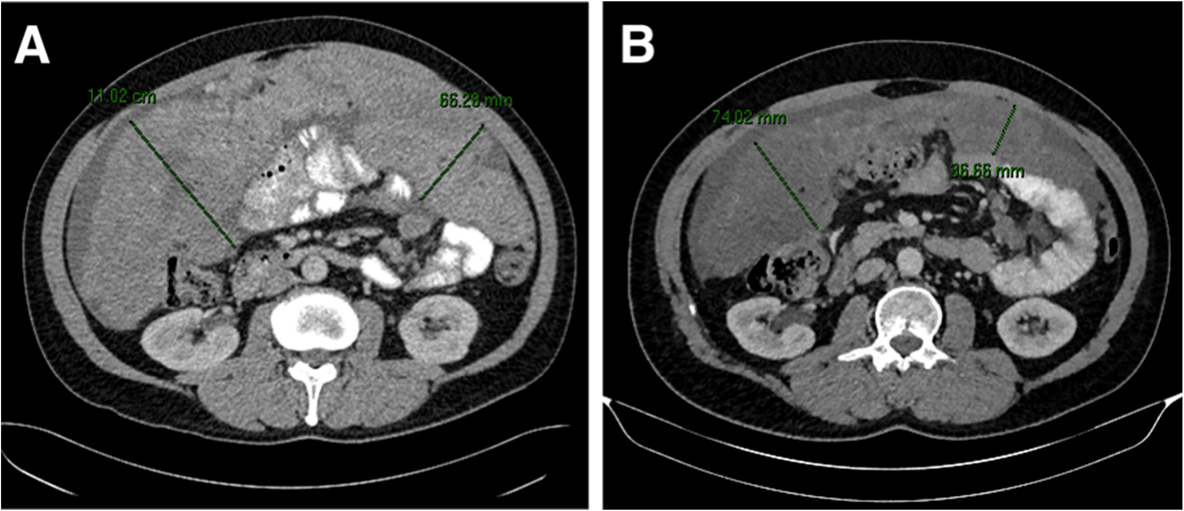
Shrinkage of peritoneal disease after 12 months of treatment with pazopanib (August 2012, A; July 2013, B) in a 30 years old man, PS:0, affected by widespread intrabdominal DSRCT with liver and soft tissue metastases.

In our series, all the patients included were males and most of them (88%) had a good ECOG PS (0–1). The high percentage of ECOG PS 0–1 in the context of second line or beyond highlights the non-occasional discrepancy between symptoms and tumour burden in this disease. It is indeed remarkable that some patients can be asymptomatic while having significant tumour load. Pazopanib was found to be well tolerated and most of the toxicities recorded were mild and manageable with medical treatment. One third of the patients (3/9) required dose reduction. In one case it was needed because of persistent fatigue: interestingly, fatigue was reported to occur in 49% of the patients included in the PALETTE placebo arm, suggesting that it might be partly due to the disease itself [6]. In one case, the dose was reduced for uncontrolled hypertension, and in the third case because of persistent G3 increase in liver enzymes, which finally led to treatment discontinuation. The outcome in this population was also encouraging: 78% of the patients reported a partial response or a disease stabilisation for more than 12 weeks. Interestingly, two patients, 47 and 30 years old, PS: 1 and 0, both with widespread peritoneal sarcomatosis and liver metastases, achieved a prolonged response (16.3 and 13.1 months respectively) with a significant shrinkage of peritoneal disease (Figure 1) associated with symptoms remission (improve of pain control).

Given the good tolerability profile and the activity shown in the metastatic setting, pazopanib should be regarded as a valuable treatment option in DSRCT patients progressing after first line chemotherapy. Pazopanib administration as a maintenance treatment after induction, with the aim to prolong disease-free survival, to improve quality of life through symptoms control and to delay the need for further chemotherapy could also represent an attractive option for these patients, whose life expectancy is likely to be limited by the disease.

We are aware that the value of this study is strongly limited by the small sample size and its retrospective character. Further evaluation through a global collaborative, prospective, study in a larger cohort of patients is mandatory.

## Conclusions

This case series shows promising activity of pazopanib in DSRCT patients progressing after prior chemotherapy without considerable toxicity. International collaborative effort is needed to confirm these preliminary findings, to establish its place in the whole treatment of DSRCT, and to finally improve the outcome of patients with this incurable and miserable disease.

## Abbreviations

VEGFR: Vascular endothelial growth factor receptors; PDGFR: Platelet-derived growth factor receptors; STS: Soft tissue sarcoma; PFS: Progression-free survival; OS: Overall survival; TTP: Time to progression; HIPEC: Hyperthermic intraperitoneal chemotherapy; WAP-IMRT: Whole abdominopelvic intensity-modulated radiation therapy; DSRCT: Desmoplastic small round cell tumour; TKI: Tyrosine kinase inhibitors; PD: Progressive disease; SD: Stable disease; PR: Partial response.

## Competing interests

The authors declare that they have no competing interests.

WVDG received speaker’s fee and financial research contribution from GSK.

## Authors’ contributions

AMF, IJ, CB, LH, WVDG, SS conceived the study, and participated in its design and coordination. AMF, LH, SL, SM collected the data. AMF, CB, IJ, WVDG, JYB, SS, RD helped to draft the manuscript. CF reviewed pathology and carried out the molecular genetic tests. AMF and SL performed the statistical analysis. All authors read and approved the final manuscript.
